# Cognitive reserve and executive functions in dual task gait performance in Parkinson’s disease

**DOI:** 10.1007/s00221-024-06897-6

**Published:** 2024-07-25

**Authors:** Helena Fernández-Lago, Pere Bosch-Barceló, José Andrés Sánchez-Molina, Mira Ambrus, Dan Rio, Miguel Ángel Fernández-Del-Olmo

**Affiliations:** 1 Department of Nursing and Physiotherapy, University of Lleida, Lleida, Spain; 2 Health Care Research Group (GRECS), Lleida Institute for Biomedical Research Dr. Pifarré Foundation, IRBLleida, Lleida, Spain; 3 Grup d’Estudis Societat, Salut, Educació i Cultura, GESEC, Department of Nursing and Physiotherapy, University of Lleida, Carrer de Montserrat Roig, 2, Lleida, 25198 Spain; 4 Department of Physical Education and Sport, Faculty of Sport Sciences and Physical Education, University of A Coruña, A Coruña, Spain; 5 Research Center for Sports Physiology, Hungarian University of Sports Science, Budapest, Hungary; 6 Sports Science Research Center, King Juan Carlos University, Madrid, Spain

**Keywords:** Cognition, Gait, Cognitive reserve, Parkinson’s disease, Dual task

## Abstract

A higher level of education was correlated with less severe motor impairment in Parkinson’s Disease (PD). Nevertheless, there is limited evidence on the relationship between cognitive reserve and motor performance in complex situations in PD. To investigate the association between cognitive reserve and the dual-task gait effect in PD. Additionally, we examined the relationship between executive function, clinical and sociodemographic variables and, dual-task gait effects. We conducted a cross-sectional study with 44 PD participants. We evaluated dual-task effect on cadence, stride length, and gait velocity. Dual-task effects were correlated with neurophysiological factors, including cognitive reserve (Cognitive Reserve Index Questionnaire), overall cognitive performance of executive functions, a specific executive function domain (Trail Making Test), and the global cognitive status (Montreal Cognitive Assessment and Mini-Mental State Examination). Age, gender, and disease severity were considered as variables to be examined for correlation. We found that cognitive reserve did not influence gait performance under dual-task conditions in this sample. However, executive functions, age, and disease severity were associated with the dual-task effect on gait. The overall cognitive performance with respect to the Trail Making Test showed an inverse relationship in the dual-task gait effect on cadence. Our study’s findings have important implications for understanding the association between executive functions, age, and disease severity with the dual-task effect on gait in PD. Pre-life factors, such as education, occupation, and leisure activity, did not contribute to coping with complex gait situations in PD.

## Introduction

Cognitive dysfunction commonly occurs in people with Parkinson’s disease (PwPD), with up to 60% of individuals developing dementia within 12 years after the diagnosis (Buter et al. 2008). In recent years, scientific literature has shown a growing interest in the discordance between the degree of neurological damage and its clinical manifestations: the same amounts of damage do not correspond to similar levels of symptomatology in patients (Stern 2009). The Cognitive Reserve (CR) concept can justify this discordance, suggesting that the brain actively attempts to combat brain damage, either by utilizing pre-existing cognitive processes or by recruiting compensatory mechanisms (Stern 2009).

A recent review demonstrates that factors, such as age and educational level influence the occurrence of Mild Cognitive Impairment (MCI) in PwPD (Baiano et al. 2020), aligning with the CR paradigm. The educational level, the most commonly used proxy for CR, correlates with better performance on neurocognitive tests sensitive to the typical frontal brain damage observed in PwPD (Ciccarelli et al. 2018). Interestingly, CR has also been associated with motor function in this population. Higher education was associated with less severe motor impairment in the Movement Disorder Society–Unified Parkinson Disease Rating Scale motor exam (MDS-UPDRS-III), a relationship explained by the protective influence of education on white matter integrity (Kotagal et al. 2015). However, a higher education level may not affect overall motor severity but could impact performance on motor tests in PwPD. Those with more CR may better utilize cognitive attention during testing, potentially improving motor speed and performance on specific tasks (Kotagal et al. 2015).

The close relationship between motor symptomatology and cognitive impairment, with its consequent decline in executive functions, is an essential aspect in the management of PwPD. In particular, the interaction between gait and a concurrent task generates a complicated situation that restricts independence and predisposes patients to falls. In 2019, Wegrzyk et al. studied the relationship between educational level and gait performance during dual-task conditions in PwPD with deep brain stimulation (Wegrzyk et al. 2019). The educational level was correlated with better gait performance during dual-task conditions compared to parameters collected before electrode implantation. However, it is essential to note that the study focused on PwPD with particular characteristics. Consequently, evidence is scarce regarding the relationship between CR and motor performance in complex situations in Parkinson’s disease (PD). Moreover, several tools have been utilized beyond the educational level to objectify CR, integrating different proxies. The multifactorial concept, like CR, encompasses many cognitive and social challenges over a lifetime. For this reason, tools such as the Lifetime of Experiences Questionnaire or the Cognitive Reserve Index Questionnaire (CRIq) have been developed and recommended (Nucci et al. 2012).

To date, no study has approached individuals with PD to assess the interaction between their gait parameters under dual-task conditions and CR, measured through tools that consider different proxies. The primary objective of this study was to investigate the association between gait performance under dual-task conditions (motor-cognitive) and CR in PwPD. We hypothesize that a higher CR is related to better gait performance in the dual-task. Additionally, we examined the relation between dual-task gait and executive functions, cognitive state and clinical and socio-demographic variables in PwPD.

## Methods

### Type of study

A cross-sectional study followed the STROBE guidelines as a quality tool.

### Participants

44 individuals diagnosed with idiopathic PD (11 females and 33 males, mean age = 63.1 ± 9.65), following the United Kingdom Brain Bank Criteria (Hughes et al. 1992), were recruited from the local PD association between 2014 and 2018. The inclusion criteria were the ability to walk for 10 min without stopping or walking assistance, absence of neurologic affectations other than PD, absence of severe visual and hearing deficits that could affect gait, and not receiving cognitive or psychological therapy. No participant showed cognitive decline as assessed by the Mini-Mental State Examination (MMSE ≤ 24) (Folstein et al. 1975; Sakurai et al. 2021; Tombaugh and McIntyre 1992), and depression as assessed by the Geriatric Depression Scale (GDS-15 > 10) (Sheikh and Yesavage 1986). At the first visit, an experienced neurologist confirmed the eligibility criteria and collected each participant’s demographic and clinical information. After the first visit, participants underwent two-day neuropsychological and gait performance evaluations by PD experts. The measurements were performed at the same time of day and ON state of medication (45 min–1 h after medication intake). All participants gave written informed consent following the declaration of Helsinki (World Medical Association, 2013) before entering the study. This study was approved by the ethical committee of the University of A Coruña (code: 2009/346).

### Variables

#### Primary variable

Dual-task effect (DTE) on stride length, cadence and gait velocity.

The participants were tested in two counterbalanced conditions: a single-task condition (walk only) and a dual-task condition (walk and perform a cognitive task). The cognitive task consisted of the phoneme monitoring paradigm, previously used in several studies of PD and gait (Connine and Titone 1996; Fernández-Lago et al. 2015; Wild et al. 2013). Participants listened to a 1-minute-long narration through wireless headphones and they had to count the times two pre-specified words were repeated in the narration. Participants were not allowed to use their voices or count on their fingers. No information about any task priority was given in the dual-task conditions.

For each condition, the participants walked up and down an 8-m walkway for 2 min at a self-selected walking velocity. Previously, the participants performed a familiarization trial during the same period and at a self-selected walking velocity. The recordings were taken in the second minute (on the straight portion, but not during the turns or each condition) using an optical detection system (Optogait, Microgait, USA).

DTE was quantified by applying the formula of Plummer and Eskes (2015), for stride length, cadence and gait velocity (Plummer and Eskes 2015).

$$\begin{gathered} DTE\, = \, \hfill \\\left( \begin{gathered} Dual\,task\,-\,single\,task\,gait\,parameter \hfill \\/single\,task\,gait\,parameter \hfill \\ \end{gathered} \right)\,\cdot\,100 \hfill \\ \end{gathered}$$

Negative DTE values indicate a reduction of the gait parameter values during the dual task when comparing to the single task, while positive DTE values indicate an increase of the gait parameters values during the dual task.

#### Neuropsychological assessments

##### Cognitive reserve index questionnaire (CRIq)

The CRIq was administered to estimate cognitive reserve. It contains 20 questions divided into three sections, addressing years of education (CRI-Education), occupation (CRI-Working Activity), and leisure activities through adulthood (CRI-Leisure). A total score is also provided (CRI-Total) by considering the three sub-scores (CRI-Leisure, CRI-Education, and CRI-WorkingActivity). All four of the resulting scores were considered for the data analysis. Higher scores mean a higher grade of cognitive reserve for participants.

##### Total executive function performance (TEFP)

Executive functions were assessed by performing several neuropsychological tasks in a computerized manner. The neuropsychological test included: Trail Making Test Part B accuracy (TMT-Bac) Trail Making Test Part B time (TMT-Bt), Digit Span Backwards (DIGSP-BW), Tower of London Movement (ToLmov), and time (ToLtime), Word Fluency (WFpf) and accuracy (WFpac), Corsi Block Test (CORSI), and Berg Card Sorting Test Errors (BCTSerr) and perseverative responses (BTCSpers). All these assessments were selected according to a meta-analysis by Kudlicka et al. (2011), as well as recommendations from a 2013 review by Dirnberger and Jahanshahi (2013) (Dirnberger and Jahanshahi 2013; Kudlicka et al. 2011). The free PEBL (The Psychology Experiment Building Language) software was used. Measures from each task were converted into z scores based on the pooled PD group, and the sign of some measures was adjusted so that higher scores indicated better performance. A single summation score was obtained from the sum of all z scores, with higher values indicating better cognitive performance. This procedure reduced the number of statistical comparisons and the role of chance. The following formula was applied to obtain the variable TEFP, to aggregate the neuropsychological data on executive functions:

$$\eqalign{& TEFP\, = \,TMT - Bac\, + \,DIGSPN - BW{\rm{ }}\, \cr & + \,WFpf\, + \,WFpac\, + \,CORSI\,-\,ToLmov \cr & {\rm{ }} - {\rm{ }}ToLtime\, - \,BCTSerr\, - \,BCTSpers\,-\,TMT\, - \,Bt \cr}$$

##### Cognitive flexibility

We separately measured the performance on Trail Making Test Part B (TMT-B) due to its contribution to predicting of the ability to complete instrumental activities of daily living in PD (Higginson et al. 2013). The TMT-B is an open-access neuropsychological test that alternates numbers and letters, requiring the patient to switch between numbers and letters in consecutive order. The TMT-B scored the accuracy (TMT-Bac) and completion time (TMT-Bt).

##### Global cognitive status

The MMSE and the Montreal Cognitive Assessment Scale (MoCA) assessed the cognitive state. The MMSE is a brief cognitive test used extensively in clinical with a maximum score of 30 points and a cut-off score of 24 or less, which detects cognitive impairment (Sakurai et al. 2021; Tombaugh and McIntyre 1992). It covers cognitive functions, including orientation, registration, attention, recall, and language. The MoCA is a cognitive test assessing cognitive functions, including short-term memory recall, visuospatial abilities, executive function, verbal abstraction, attention, concentration, working memory, language, and orientation. A cut-off score of 25 or less on the MoCA indicates possible mild cognitive impairment (MCI), and the maximum score is 30 (Nasreddine et al. 2005).

#### Clinical and socio-demographic variables

We registered the variables of age, gender, disease duration, and disease severity. The severity of the disease was measured with the Hoehn and Yahr scale (H&Y), the Movement Disorder Society–Unified Parkinson Disease Rating Scale (MDS-UPDRS) and UPDRS motor score (MDS-UPDRS-III) (Goetz et al. 2008).

### Statistical analysis

The descriptive analysis includes absolute and relative frequencies of qualitative and mean variables (standard deviation) and median (percentiles 25 and 75) of quantitative variables. To estimate whether the differences between single and dual-task performance for each gait parameter were significant, the t-test was used on each pair of gait variables. The principal analysis of the DTE (DTE Velocity, DTE Cadence and DTE Stride Length) includes Pearson correlations, estimating the importance of potentially explanatory variables through the Boruta algorithm with all variables (CRI-Total, CRI-Leisure, CRI-Education, CRI-WorkingActivity, TEFP, TMT-Bac & TMT-Bt, MMSE, MoCA, H&Y, MDS-UPDRS & MDS-UPDRS-III, gender, age, disease duration), and, in a second version, with the less critical variables omitted to increase the cases to be analyzed by the algorithm. Finally, for each main variable, a multivariate linear regression model is studied with the variables not rejected by Boruta’s algorithm as candidates to be part of the model and maintaining only those demonstrating a statistically significant contribution to variance analysis. Multicollinearity was estimated by using the Variance Inflation Factor (VIF) in all multivariate models. Details on variables included for each regression model, as well as their coefficients can be seen on Table 3 (MDS-UPDRS-III, MDS-UPDRS, TEFP, log(TMT_Bt), Age). The coefficient of determination is proportional to the percentage of variability estimated by the final model. Outlier values were validated and included into the analysis. Normality and homoscedasticity of the residuals of the models was checked. Only once with the TMT-Bt variable was heteroscedasticity observed, leading to a logarithmic transformation. Statistical analysis was carried out using the R (R Core Team 2023) program and applying a level of statistical significance of 0.05.

## Results

Table 1 summarizes the clinical, socio-demographic, and neuropsychological variables. One participant did not complete MDS-UPDRS-III. Six participants did not complete the TEFP measurements. 31 participants showed a scored ≤ 25 in MoCA.

**Table 1:** Mean and standard deviation of clinical and socio-demographic and neuropsychological variables

	Mean (SD)
Age (yr)	63.1 (9.65)
Height (m)	166 (9.85)
Disease duration (yr)	6.25 (4.44)
MDS-UPDRS-III	14.9 (7.00)
MDS-UPDRS	37.5 (13.5)
H&Y	1.93 (0.35)
PDQ-39	23.3 (17.4)
MMSE	28.2 (1.72)
MoCA	24.8 (3.26)
TEFP	0.69 (4.54)
TMT-B ac	0.91 (0.06)
TMT-B t (sec)	323 (142)
CRI-Total	107 (19.0)
CRI-Education	100 (16.9)
CRI-WorkingActivity	106 (14.8)
CRI-Lesiure	109 (20.4)
	**Frequencies**
Gender M	33 (75.0%)
H&Y: 1	*n* = 4 (9.09%)
H&Y: 1.5	*n* = 2 (4.55%)
H&Y: 2	*n* = 34 (77.3%)
H&Y: 2.5	*n* = 4 (9.09%)
CRI: Low/Medium-low	5 (11.4%)
CRI: Medium	24 (54.5%)
CRI: Medium-high/High	15 (34.1%)

Yr = years; m = metres; MDS-UPDRS-III = Movement Disorder Society–Unified Parkinson Disease Rating Scale motor score; MDS-UPDRS = Movement Disorder Society–Unified Parkinson Disease Rating Scale; PDQ-39 = Parkinson Disease Questionnaire 39; H&Y = Hoen and Yahr; M = Male; F = Female; MMSE = Mini-Mental State Examination; MoCA = Montreal Cognitive Assessment Scale; TEFP = Total Executive Function Performance; TMT-Bac = Trail Making Test Part B accuracy, TMT-Bt = Trail Making Test Part B time; sec = seconds, CRI = Cognitive Reserve Index

### Differences between single and dual-task performance for all gait variables

We found statistically significant differences for all three gait parameters (*p* < 0.05) when comparing the subtraction of single-task values from dual-task values. Stride length was higher during single-task than dual-task, with an average of 4.04 cm difference (*p* < 0.0001; CI 2.75,5.34). Cadence was also higher during single-task than dual-task, with an average of 0.02 Hz difference (*p* < 0.0001; CI 0.01,0.04). Gait velocity was higher during a single task than a dual task, with an average of 0.07 m/s difference (*p* < 0.0001; CI 0.05,0.09). Implementing the dual task resulted in an overall reduction in all gait parameter values as calculated with the DTE formula. Table 2 details the mean difference, confidence interval, and value of significance of single and dual-task gait parameters comparisons and the dual-task effects variables.

**Table 2:** Gait parameters and dual-task effect

Gait parameter	Mean ST (SD)	Mean DT (SD)	Difference mean (ST-DT)	P value	95% CI	DTE (SD)
Stride Length (cm)	125 (20.3)	121 (21.5)	4.04	< 0.0001*	[2.75, 5.34]	-3.51 (3.96)
Cadence (Hz)	0.95 (0.08)	0.93 (0.08)	0.02	< 0.0001*	[0.01, 0.04]	-2.40 (3.92)
Gait Velocity (m/s)	1.19 (0.22)	1.12 (0.23)	0.07	< 0.0001*	[0.05, 0.09]	-5.97 (6.04)

ST = single task condition; DT = dual task condition; DTE = dual task effect; cm = centimeters; Hz = hertz; m/s = meters/seconds; SD = standard deviation; CI = Confidence Interval; *p < 0.05

### Results of the DTE associations

Information on the regression models used for all gait parameters and DTE can be found on Table 3.

**Table 3:** Models of dual-task effects (DTE) on stride length, cadence and velocity

Model	Adjusted R2 (%)	Variable	Beta	95% CI	p-value
DTE on Stride Length	7.05	MDS-UPDRS-III	-0.16	[-0.33, 0.02]	0.075
DTE on Cadence	36.55	TEFP	0.55	[0.22, 0.88]	0.002*
		MDS-UPDRS	-0.10	[-0.18, -0.02]	0.018*
		log(TMT_Bt)	4.1	[0.32, 7.9]	0.034*
DTE on Velocity	19.54	Age	-0.22	[-0.39, -0.04]	0.019*
		MDS-UPDRS-III	-0.26	[-0.51, -0.01]	0.039*

DTE = Dual-task effect; MDS-UPDRS-III = Movement Disorder Society–Unified Parkinson Disease Rating Scale motor score; TEFP = Total Executive Function Performance; MDS-UPDRS-III = Movement Disorder Society–Unified Parkinson Disease Rating Scale; log(TMT_Bt) = logaritmic transformation of Trail Making Test Part B completion time; CI = Confidence Interval; *p < 0.05. For all explanatory variables and in all multivariate models, the Variance Inflation Factor (VIF) was less than 1.8

#### DTE - stride length

There was no variable that significantly explained the variability of DTE for stride length. Despite this, MDS-UPDRS-III explained 7.05% of the variability with a negative association (β = -0.16). The estimated associative change at each MDS-UPDRS-III unit was − 0.155, which did not reach statistical significance (p-value = 0.075).

#### DTE - cadence

TEFP, TMT-Bt, and MDS-UPDRS explained 36.55% of the variability in DTE for cadence. The multivariate regression model showed that the estimate of the change associated with a 1-unit difference in TEFP was significantly positive (β = 0.55, 95% CI = [0.22–0.88], p-value = 0.002). A higher score in TEFP (a better global cognitive performance) was associated with higher values in DTE, i.e., a higher cadence under the dual-task condition, and vice versa.

The multivariate regression model showed that the estimate of the change associated with a 1-unit difference in logTMT-Bt unit was significantly positive (β = 4.1, 95% CI = [0.32, 7.9], p-value = 0.034). A higher time consumption in logTMT-Bt (poor execution) was associated with higher values in DTE, i.e., an increment in cadence under the dual-task condition.

The multivariate regression model showed the estimate of the change associated with a 1-unit difference in MDS-UPDRS was significantly negative (β = -0.10, 95% CI = [-0.18, -0.02], p-value = 0.018). A higher score in MDS-UPDRS (i.e., more severity of the disease) was associated with lower values in DTE, i.e., a reduction in cadence under the dual-task condition.

#### DTE - velocity

The Boruta algorithm confirmed the relationship with the TEFP, which was examined in the multivariate model alongside age (negatively correlated with RCT, with *r* = -0.62) and MDS-UPDRS-III. The multivariate regression model showed that the TEFP contribution to the model was not significant (p-value = 0.6446), even when Age and MDS-UPDRS-III were already included.

The relationship between age and UPDRS-III accounts for 19.54% of the variability. The estimate of the change associated with a 1-unit difference in Age was significantly negative (β = -0.22, 95% CI = [-0.39, -0.04], p-value = 0.019) (Fig. 4). Likewise, the estimate of the change associated with a 1-unit difference in UPDRS-III was significantly negative (β = -0.26, 95% CI = [-0.51, -0.01], p-value = 0.039).

## Discussion

The primary goal of this cross-sectional study was to examine the relationship between cognitive reserve (CR) and dual-task walking performance in PwPD. Our findings, which provide novel insights, revealed that CR, as measured by the Cognitive Reserve Index Questionnaire (CRIq), did not significantly impact gait performance during dual-task conditions in this sample. Instead, executive function performance influenced cadence and gait velocity under dual-task conditions in our sample. Age and disease severity were also associated with gait performance during dual-task conditions.

Our sample’s walking pattern under dual-task conditions was characterized by slower velocity and a shorter stride length (resulting in a dual-task cost), which aligns with previous research (De Freitas et al. 2020; Rochester et al. 2004). Gait velocity, stride length and cadence all showed significative differences when comparing ST and DT walking. In average, stride length in DT was 4.04 cm lower, which is above clinically relevant differences of 3.6 cm (Baudendistel et al. 2024). Older age and higher disease severity were associated with poorer gait performance during dual-tasking. Nevertheless, our findings showed no significant impact of CR on motor performance under complex tasks. In contrast, a recent cross-sectional study found an association between the CRIq-Total score and overall motor function, as assessed by MDS-UPDRS-III. This protective impact of CR was more pronounced in PwPD with a moderate to extended disease duration (> 9 years) than less disease duration. Furthermore, they noted that the influence of CR exhibited variations between those with low CRIq scores and those with higher CRIq total scores (Guzzetti et al. 2019). All PD participants in our study had a disease duration of less than 9 years, and only 11.4% displayed a low CRIq. As a result, the characteristics of our sample may need to be more representative to demonstrate the role of CR in the dual-task effect on gait in PwPD. The impact of CR on preserving motor function may only be partially noticeable in the early stages but could become more significant as the disease advances. Moreover, among the 44 PD participants of our study, 31 displayed MCI (scoring ≤ 25 on the MoCA test). Given that higher CR has been linked to a reduced risk of the gradual progression of MCI in PwPD (Gu and Xu 2022), it would be advisable for future studies to include diverse samples with varying disease durations and levels of cognitive reserve (high, medium, and low). Furthermore, prospective evaluations should be conducted precisely in PwPD with MCI.

Our results indicated that executive function performance influenced gait cadence under dual-task conditions in PwPD. These results align with previous research in which PwPD exhibited a slower velocity, shorter stride length, and reduced gait cadence; this gait pattern has been suggested to be indicative of PwPD of this sample prioritizing safe walking over cognitive performance, as a consequence of the cognitive load posed by the dual task (Penko et al. 2018) and that differs from PwPD with more severe cognitive impairment who may adopt a posture-second prioritization strategy (Johansson et al. 2021). Finding a balance between cadence and stride length is crucial to walking faster and effectively. Cho et al. (2010) proposed that difficulty synchronizing gait velocity and cadence is a primary issue in PD gait (Cho et al. 2010). However, no association between cognitive function and velocity and stride length DTE was found in our sample. In low-severity populations Levodopa still has a strong effect in regulating gait parameters compared to more advanced patients, especially on stride length and velocity (Cho et al. 2010; Curtze et al. 2015). This may justify why our low-severity sample did not present a relation between cognitive function and DTE on gait velocity and DTE on stride length, since attentional resources are not as demanded due to the present effect of Levodopa.

Interestingly, the executive function performance tests used in this study exhibited an inverse relationship with each other. Walking at a lower cadence under dual-task conditions was associated with lower overall TEFP, including several neuropsychological executive functioning tests. On the contrary, a prolonged execution time on the TMT-B, indicating poorer test performance, was associated with walking at a higher cadence under dual-task conditions. The connection between dual-task gait impairments in PwPD and a specific cognitive domain remains uncertain whether these deficits originate from a targeted cognitive function or a broader decline in executive function (Penko et al. 2018). Our study helps bridge this gap by suggesting that the TMT-B may be more specific in addressing the distinct gait impairments observed in PD than cognitive tests that measure executive functions globally. The TMT-B is also multifactorial, encompassing cognitive aspects like visual scanning, sequencing, and cognitive flexibility. The sequencing component of the TMT was revealed as a significant factor in predicting activities of daily living in PD (Higginson et al. 2013). Consequently, sequencing more steps may increasedifficulty for individuals with PD in dual-task conditions.

### Strengths and limitations

The current study enhances our understanding of the cognitive aspects of everyday physical tasks and helps guide the customization of rehabilitation for specific patient profiles. However, our study has several limitations. First, it is part of a larger project focused on training cognitive and motor skills in PwPD, which influenced the sample selection and the design of this sub-study to understand CR. Secondly, no power analysis was performed: we aimed to explore which patients’ characteristics were related to the variability in dual-task gait performance. In our sample of 44, the adjusted regression models had no more than three variables, and therefore more than 15 subjects per variables, which is considered enough for adequate estimation of regression coefficients, standard errors and confidence intervals (Austin and Steyerberg 2015). Nevertheless, despite not meeting the initial hypothesis, the findings obtained can contribute to a deeper interpretation of the interactions between dual-tasking and CR, considering the sample’s characteristics and guiding future research steps. Third, there were numerous missing values for the TEFP measure compared to the other cognitive variables, which could potentially obscure any stronger correlations it may have with other gait parameters. Still, the TEFP outcome used in this study integrates several neuropsychological executive tests in a comprehensive manner, which aids in more accurately identifying the influence of executive function on dual-task performance in PD than global cognitive status tests, such as MoCA. Finally, we recommend that future studies consider other cognitive tasks to accommodate different levels of cognitive demand.

## Conclusions

Pre-life experiences, such as education, occupation, and leisure activity, did not influence coping with dual-task gait in individuals with PwPD. Executive function performance, age, and disease severity influenced walking patterns while performing a concurrent cognitive task. We suggest that future research related to cognitive reserve should incorporate representative samples focusing on PD and mild cognitive impairment. Additionally, future studies should investigate the contribution of specific cognitive domains in dual-task gait impairments in PD.

## Data Availability

Study data are available upon request.
